# Supplementary figures and images for: High-throughput expansion microscopy enables scalable super-resolution imaging
Source: eLife. 2024 Nov 26;13:RP96025. doi: 10.7554/eLife.96025 (PMC11594540; doi:10.7554/eLife.96025)

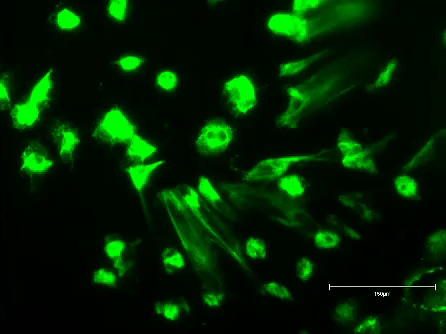

Supplement: Supplementary file 1 [file elife-96025-fig1-video1.gif]

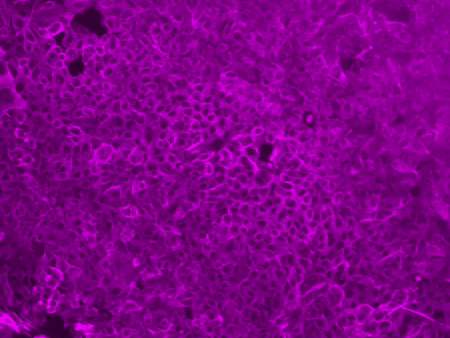

Supplement: Supplementary file 2 [file elife-96025-fig1-video2.gif]

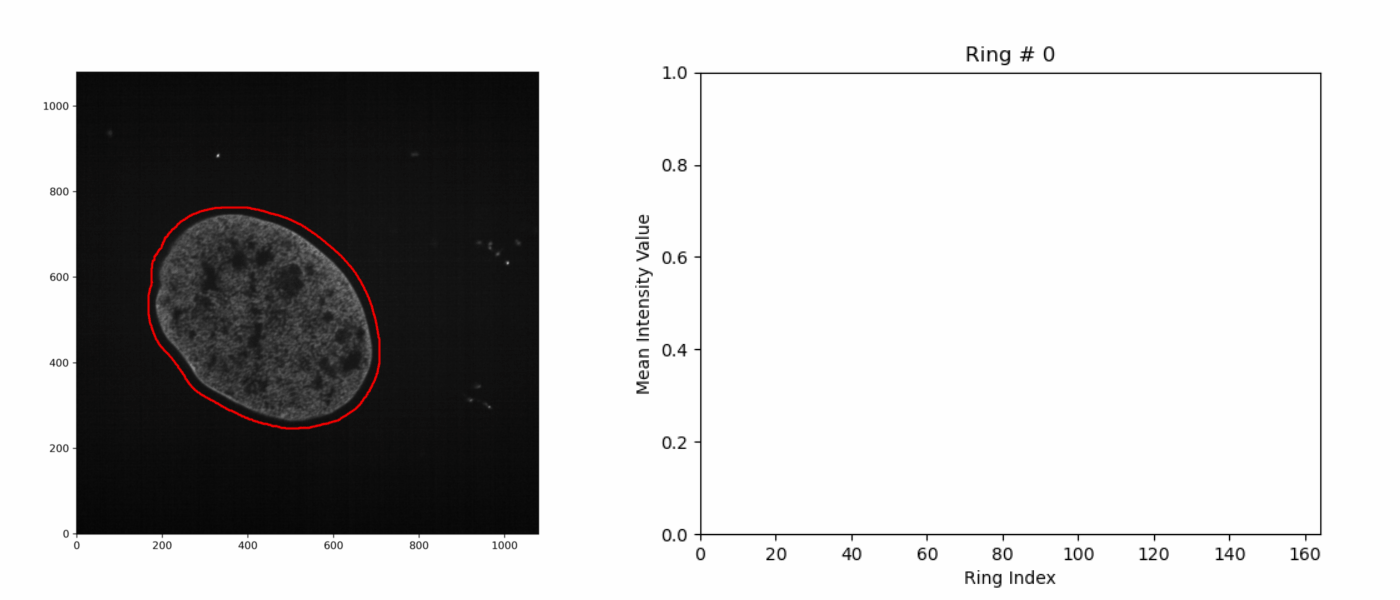

Supplement: Supplementary file 3 [file elife-96025-fig3-video1.gif]
